# Gait biomechanics in patients with intra-articular tibial plateau fractures – gait analysis at three months compared with age- and gender-matched healthy subjects

**DOI:** 10.1186/s12891-021-04577-y

**Published:** 2021-08-17

**Authors:** Anna Fändriks, Roy Tranberg, Jón Karlsson, Michael Möller, Roland Zügner

**Affiliations:** 1 Department of Orthopaedics, Institute of Clinical Sciences, Sahlgrenska Academy, University of Gothenburg, Gothenburg, Sweden; 2 Department of Orthopaedics, Institute of Clinical Sciences, Sahlgrenska Academy, University of Gothenburg, Sahlgrenska University Hospital, SE-41345 Gothenburg, Sweden

**Keywords:** Fracture, Tibia, Gait analysis

## Abstract

**Introduction:**

Tibial plateau fractures involve the knee joint, one of the most weight-bearing joints in the body. Studies have shown that gait asymmetries exist several years after injury. Instrumental gait analysis, generating both kinematic and kinetic data from patients with tibial plateau fractures, is uncommon.

**Aim:**

To examine walking ability and knee range of motion in patients suffering intra-articular tibial plateau fractures.

**Method:**

Twenty participants, eight males and 12 females, aged 44 years (range 26–60), with unilateral isolated tibial plateau fractures, were examined 12 weeks (range 7–20) after injury. The investigation consisted of passive range of motion (ROM) using a goniometer, six-minute walking test (6 MW), pain estimation using the visual analogue scale (VAS), the “Knee injury and Osteoarthritis Outcome Score” (KOOS) self-assessment questionnaire and instrumental 3-dimensional gait analysis (3DGA). 3DGA included spatiotemporal variables (speed, relative stance time, step length), kinematic variables (knee flexion, knee extension, ankle dorsiflexion) and kinetic variables (generating knee power (extension) and ankle power (plantarflexion)). A skin marker model with twenty reflective markers was used. Non-parametric tests were used for comparisons of the injured leg, the uninjured leg and a reference group.

**Result:**

The participants walked more slowly compared with healthy references (*p* < 0.001). Stance time and step length was shorter for the injured side compared with the uninjured side (*p* < 0.014). Step length was shorter compared with the reference group (*p* = 0.001). The maximum knee extension in the single stance phase was worse in the injured side compared with the uninjured side and the reference group (*p* < 0.001) respectively. The maximum ankle dorsiflexion during stance phase was higher in the injured leg compared with the uninjured side and the reference group (*p* < 0.012). Maximum generated power in the knee was lower in the injured side compared with the uninjured side and the reference group (*p* < 0.001 respectively). The same was true of maximum power generated in the ankle (*p* < 0.023). The median KOOS value was lower in the study group (*p* < 0.001). ROM showed decreased flexion and extension in the knee joint and decreased dorsiflexion in the ankle joint compared with the uninjured side (*p* < 0.006). The average distance in the six-minute walking test was shorter in the study group (*p* < 0.001).

**Conclusion:**

Patients who have sustained tibial plateau fractures generally display a limitation in their walking pattern 3 months after injury. These limitations are mainly related to the inability to extend the knee.

## Background

Tibial plateau fractures (TPF) are fractures of the proximal tibia, involving the articular surface and the metaphyseal area [1]. According to recent Swedish and Scottish studies, TPF accounts for 1–2% of all fractures [2, 3]. TPF frequently occur after high-energy trauma to the knee, such as skiing or a collision between a car and a pedestrian or cyclist, but also after low-energy trauma, in the osteoporotic patient [4, 5].

There are a limited number of studies that describe the rehabilitation process after a TPF. Early range of motion training and specific isometric muscle activation and training of gait function starts as soon as possible post-operatively [6–9]. Previous studies report that patients are allowed to partially weight-bear or even be treated with non-weight-bearing for a period of up to 16 weeks, depending on the severity of the fracture [6, 7, 9, 10]. Some fractures are treated with a knee brace, even though there is limited evidence of the effect of a knee brace [8–10].

The outcomes after treatment vary considerably in terms of knee laxity, restoration of range of motion and radiological outcome. According to Rohra et al. patients with high energy fractures had excellent to good outcomes when assessed with radiological outcomes (Rasmussen modified score) and patient reported outcome (Oxford Knee Score) [7]. Similar results are presented by Giannotti et al., who assessed TPF-patients with Knee Society Score including range of motion, knee laxity, pain, walking and stair climbing as well as Rasmussen Score. However, at arthroscopy due to removal of fixation material, it was shown that all participants had chondral damage [11]. The results from these studies have been shown to be satisfactory, with only minor remaining problems for the patient [7, 11]. There are, however, other studies reporting that complex fractures of the tibial plateau result in long-term disability, with limited range of motion, activity-related pain and knee instability [6, 9, 12].

Clinical assessments normally include measurement of range of motion, observational gait analysis and sometime some kind of functional test such as getting up from a chair. Three dimensional gait analysis (3DGA) is a recent method to examine dysfunction and gait asymmetries. The advantage of 3DGA is that it provides a more precise and objective examination and generates data related to spatiotemporal parameters, joint angles (kinematics) and forces (kinetics) during walking in three dimensions [13]. The disadvantage is that it can be considered time consuming and costly.

A few studies have previously reported on gait biomechanics after a TPF. According to Warschawski et al., spatiotemporal parameters, such as speed, cadence, step length and stance time, can be affected up to several years after the injury [14]. Delenau et al. examined patients who were treated surgically three, six and 12 months after injury and noted improved cadence, step time, gait cycle length and knee flexion over time. Patients with more severe fracture types had poorer outcomes [15].

There are only a limited number of studies based on patient-reported outcomes and gait analysis after a TPF. To our knowledge, there are no previous studies of gait analysis with kinematic and kinetic variables.

The aim of the study was to investigate the gait function and joint mobility in patients with intra-articular tibial plateau fractures 3 months after injury.

The hypothesis was that the walking pattern was affected in patients suffering a TPF compared with a reference group and that patients would have less joint mobility and more pain than a reference group.

## Method

### Subjects

The selection of study participants was made using the Swedish Fracture Register (SFR) [16]. Classifications of tibia fractures in the SFR have shown good reliability [17]. Patients with a TPF (ICD-code S82.10) [18] registered at Sahlgrenska University Hospital were assessed for participation in the study. The inclusion criteria were ICD-10-code S82.10, age 18–65 years and a unilateral TPF type B or C according to the Arbeitsgemeinschaft für Osteosynthesefragen/Orthopaedic Trauma Association (AO/OTA) classification [19]. The exclusion criteria were as follows; patients with multiple fractures, on-going treatment with an external fixator, previous injury or surgery to the lower extremities such as arthroplasty in the lower extremity or other previously diagnosed impact on gait function for example a neurological disorder. Patients with cognitive disorders were also excluded because they may have difficulty to follow instructions and cooperate during the examination process. Type A-fractures are extraarticular fractures of the proximal tibia, and thus usually do not have restrictions about weight -bearing and were therefore excluded. Multiple fractures usually have more restrictions and this could possibly affect the neuromuscular pattern even more. At the start of the study, a study group without any confounding factors was desired, which is why these exclusion criterias were chosen.

An instrumental gait analysis and clinical examination were scheduled 2 weeks after patients were able to walk 15 m without walking aids (Table 1). At this time, the patients had revisited the orthopaedic surgeon and had no weight-bearing restrictions.

**Table 1:** Demographic data for the study population and reference groups 1 and 2

	Study population		Reference group 1		Reference group 2	
	Median	Range	Median	Range	Median	Range
Gender male/female	8/12	–	8/12	–	8/12	–
Age, years	44.5	26–62	45	25–63	45.5	26–60
Height, m	1.70	1.54–1.94	1.74	1.62–1.91	1.75	1.51–1.96
Weight, kg	72	60–106	71	57–91	68	51–110
Affected side left/right	16/4	–	–	–	–	–
Non-surgical/surgical treatment	4/16	–	–	–	–	–
Number of days from injury to gait analysis	85	51–140	–	–	–	–
Number of days without walking aids before gait analysis	14	8–42	–	–	–	–
Fracture type (AO/OTA)						
*41-B1.1, N*	3		–		–	
*41-B1.3, N*	1		–		–	
*41-B2, N*	5		–		–	
*41-B3, N*	5		–		–	
*41-C1, N*	1		–		–	
*41-C3, N*	5		–		–	

**Fig. 1:**
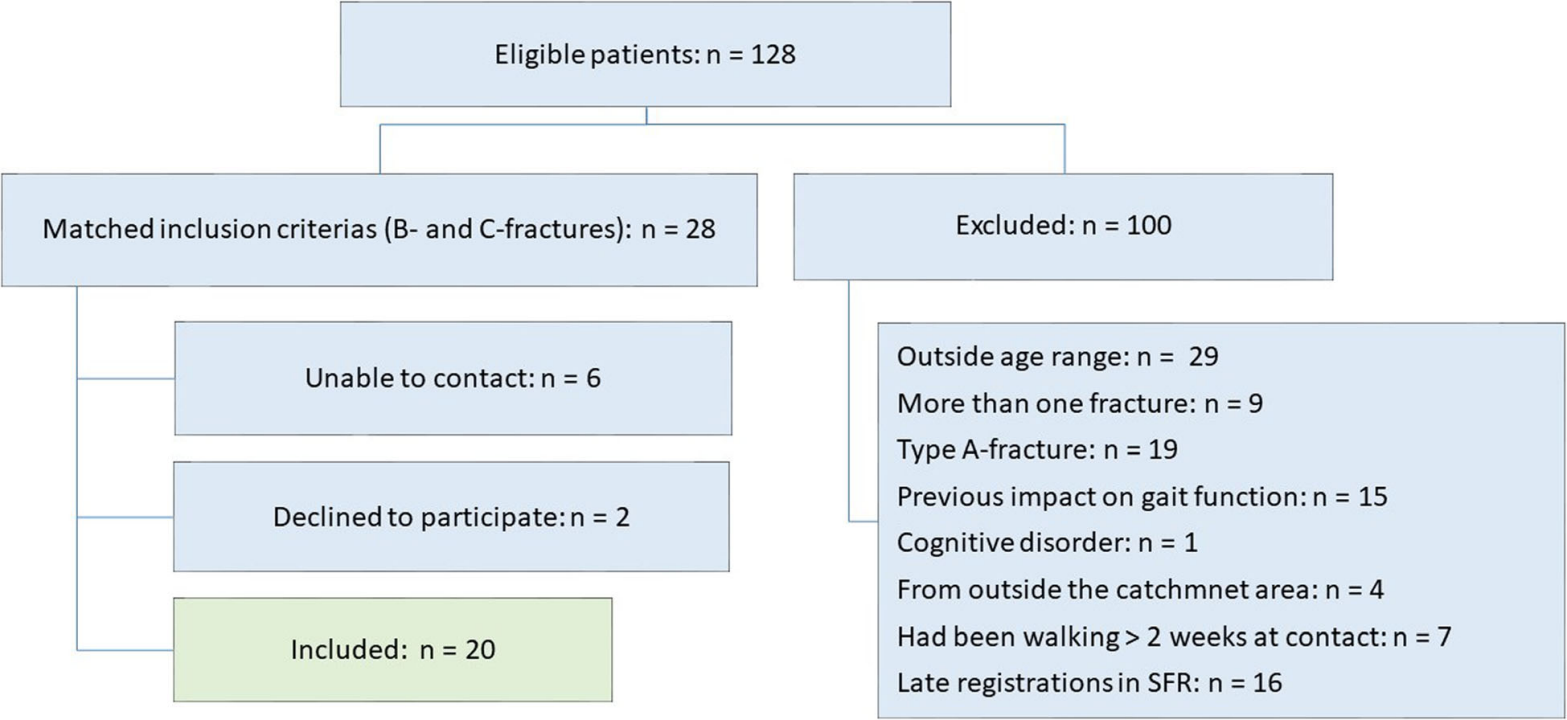
Flow chart for inclusion and exclusion of participants

During the periods February–April 2018 and June 2018–March 2019, 128 eligible patients with fractures to the upper end of the tibia were registered. One hundred patients did not meet the inclusion criteria. Of the remaining 28 patients, eight declined to participate or could not be contacted. Twenty patients were included in the study (Fig. 1).

There are two different reference groups in this study (Table 1). Reference group 1 is the control group for the instrumental gait analysis and consisted of individuals who are included in the gait laboratory’s database. Reference group 2 consisted of new material for this study and served as control group for the six-minute walking test and self-assessment questionnaire. Both groups are gender and age matched (+/− 4 years). Before participation, the individuals were verbally asked about their health status with questions concerning pain, previous injuries and if they felt completely healthy in the lower extremities. Only individuals without previous knee problems were included in the study as controls.

### Data collection

All the examinations were carried out by certified physiotherapists at the instrumental gait laboratory, *Orthopaedic Research Unit* at *Sahlgrenska University Hospital/Mölndal*. The evaluation tool and survey instrument presented in this study were conducted in the following order.

*Self-assessment questionnaire:* the Swedish version of the Knee injury and Osteoarthritis Outcome Score (KOOS) was used [20]. This study presents the KOOS in the five domains and also as the average of all five domains. No previous reliability and validity studies have been performed for patients with TPF, but the KOOS has shown high test-retest reliability and is considered to have good validity in patients with knee osteoarthritis [21].

**Fig. 2:**
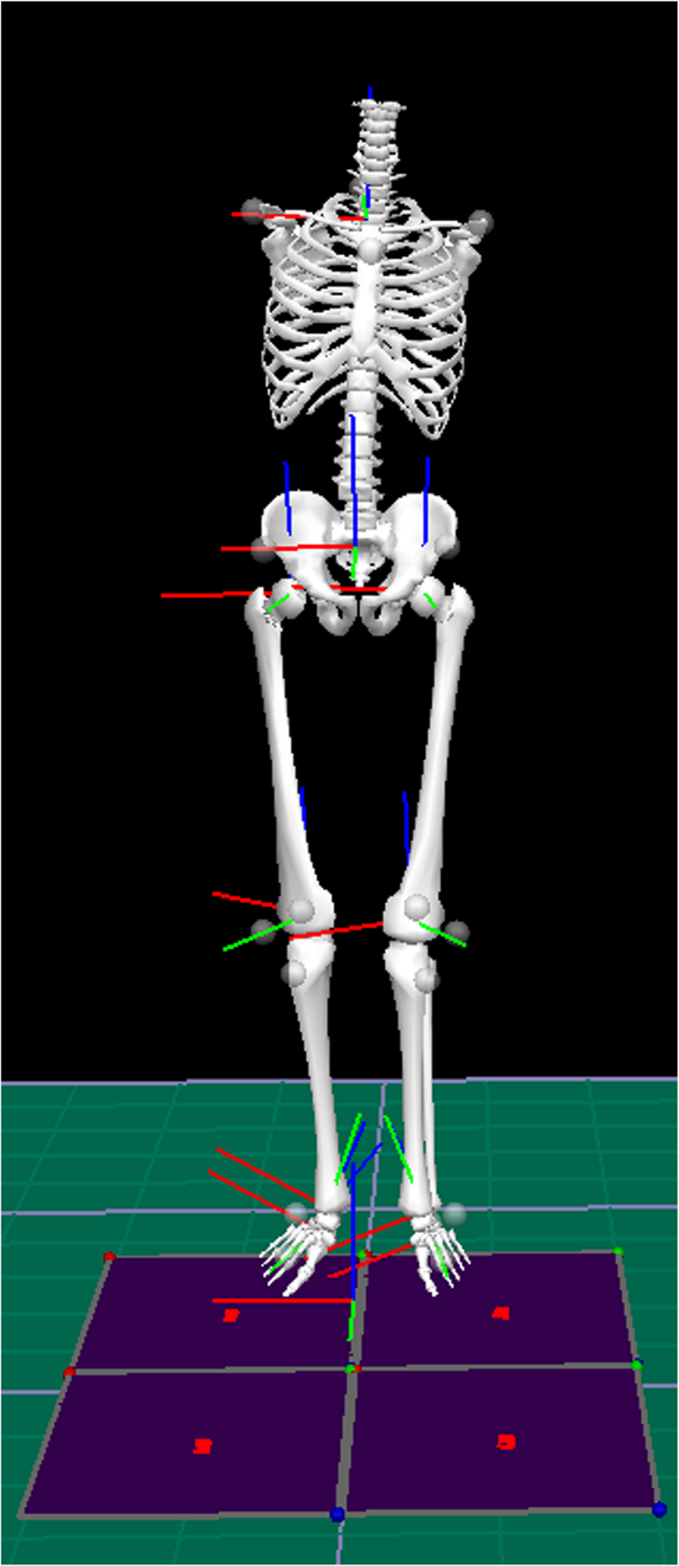
Skin-marker model

*Instrumental gait analysis:* in the instrumental gait analysis, 16 high-speed video cameras (Qualisys 7+, Qualisys AB, Göteborg, Sweden) and four force plates (Amti Optima OPT, Watertown, MA, USA) were used. The camera system was calibrated before data registration and the calibrated measurement volume was 60 m^3^ (6 × 4 × 2.5 m). Recording frequency was set at 240 Hz. Twenty reflective markers were attached to the skin with double-sided tape according to a marker model described by Weidow et al. and Zügner et al. [22, 23]. The marker model is based on anatomically well-defined reference points (Fig. 2). Three reflective markers were attached to each foot (tuber calcanei, lateral malleoli and between the second and third metatarsal bone). Three reflective markers were attached to each knee (lateral knee joint line, proximal boarder of the patellae and tibial tubercle). Three reflective markers were attached to the pelvis (sacrum and spina iliaca anterior superior bilaterally) and five reflective markers were attached to the trunk (Th12, Th2, acromion bilaterally and manubrium sterni) [22, 23].

The 3D-markers’ positions are both used for defining the size and the orientation of each body segment and for tracking of segments respectively. Inter-segment joint angles are calculated following a Cardan sequence of rotations, starting with flexion/extension, followed by adduction/abduction and internal/external rotation. Data obtained from the force plates, ground reaction forces (GRF) are used for calculation of joint moment and joint power. All variables were calculated using Visual 3D™ (C-Motion, Inc., Germatown, USA) software.

The following variables were analysed. The spatiotemporal variables, speed (m/s), step length (m) and relative stance time (percentage of gait cycle). Kinematic variables, in terms of degrees of flexion in the knee joint during gait cycle, extension in the knee joint during single stance and dorsiflexion in the ankle joint during stance phase. Kinetic variables during the stance phase were; generated power (W/kg) across the knee- and ankle joint respectively.

Instrumented gait analysis has shown moderate to good reliability for kinematic variables, with the best results in measurements in the sagittal plane [13, 23]. The marker model has been evaluated for the validity of kinematic variables in the knee and hip joint and has shown the best conformity for movements in the sagittal plane [24, 25]. No reliability and validity studies have been performed for patients with TPF.

All the instrumental gait analysis was performed barefoot. The walking distance for the measurement was approximately 10 m and was repeated until six approved gait trials had been recorded. An approved walking attempt meant that the study participant loaded each foot at least once on one of the force plates without touching two force plates simultaneously or touching any part of the foot outside the force plate area.

After analysing the gait files after the gait analysis, the median of approved walking attempts in the study population was six (range 2–6). Eighteen participants had six approved walking attempts and one participant had five approved walking attempts. One participant had two approved walking attempts for the kinetic variables on the injured side and six approved walking attempts for the kinematics. The median number of approved walking attempts in the reference group was six (range 3–6). Seventeen participants had six approved walking attempts, two participants had five approved walking attempts and one participant had three approved walking attempts.

*Passive range of motion (ROM):* the ROM in the knee (extension and flexion) and ankle joints (dorsiflexion) was measured with a goniometer (splint length 31 cm) with the participant lying supine on a bench [26]. The values were rounded to the nearest five degrees. Measurements with a goniometer have shown good validity and reliability in previous studies [21, 27].

*Pain estimation and six-minute walking test (6 MW):* a visual analogue scale (VAS) was used for pain estimation [28]. Participants estimated the pain before walking according to the VAS and then immediately after the test. The 6 MW was conducted in a 30-m corridor with markings every ten metres [29]. The participants were told to walk as far as possible during the space of 6 min, but they were not allowed to run.

The VAS has shown good reliability and validity when assessing pain [30]. The 6 MW is reliable for patients with hip fractures and patients treated with primary knee arthroplasty [31, 32]. Validity tests for patients who have knee osteoarthritis have also shown a correlation with endurance (maximum oxygen consumption), as well as with strength in the hamstrings and quadriceps muscles [33]. No previous reliability and validity studies have been performed for patients with TPF.

### Demographic data

Data for the study population and reference groups 1 and 2 are presented in Table 1.

### Data processing/statistical analysis

Statistical analysis was carried out using IBM SPSS Statistics 22. The data were not normally distributed according to the Shapiro-Wilks test. Wilcoxon’s signed-rank test was used to calculate differences between injured and uninjured legs and VAS differences before and after the walking test. The Mann Whitney U-test was used to calculate differences between the study population and the reference groups. The significance level was set at *p* < 0.05. Descriptive data are presented with number (N), median value and range. For the instrumental gait analysis, a median value for each participant’s approved walking attempts was calculated. The individual median values were then converted to a total median value with range for the group, which is the study result.

Post-hoc power calculations were performed on the differences from the study group and the control group for all included variables. The statistical power ranged between 38 and 99%, with two variables not reaching 90% power; stance time and knee flexion during gait cycle.

### Ethical considerations

The study has received ethical approval from the Ethics Review Board in Gothenburg (Dnr: 153–18, 2018-04-16).

## Results

Results from the KOOS self-assessment questionnaire are presented in Table 2.

**Table 2:** KOOS Results

	Study population		Reference group 2		*p*-value participants/reference group 2
	Median	Range	Median	Range	
Total score	48	17–92	100	64–100	< 0.001
Pain	75	11–100	100	75–100	< 0.001
Symptom	58	25–96	100	54–100	< 0.001
ADL	76	31–100	100	76–100	< 0.001
Sport	15	0–70	100	40–100	< 0.001
QOL	38	13–94	100	56–100	< 0.001

**Fig. 3:**
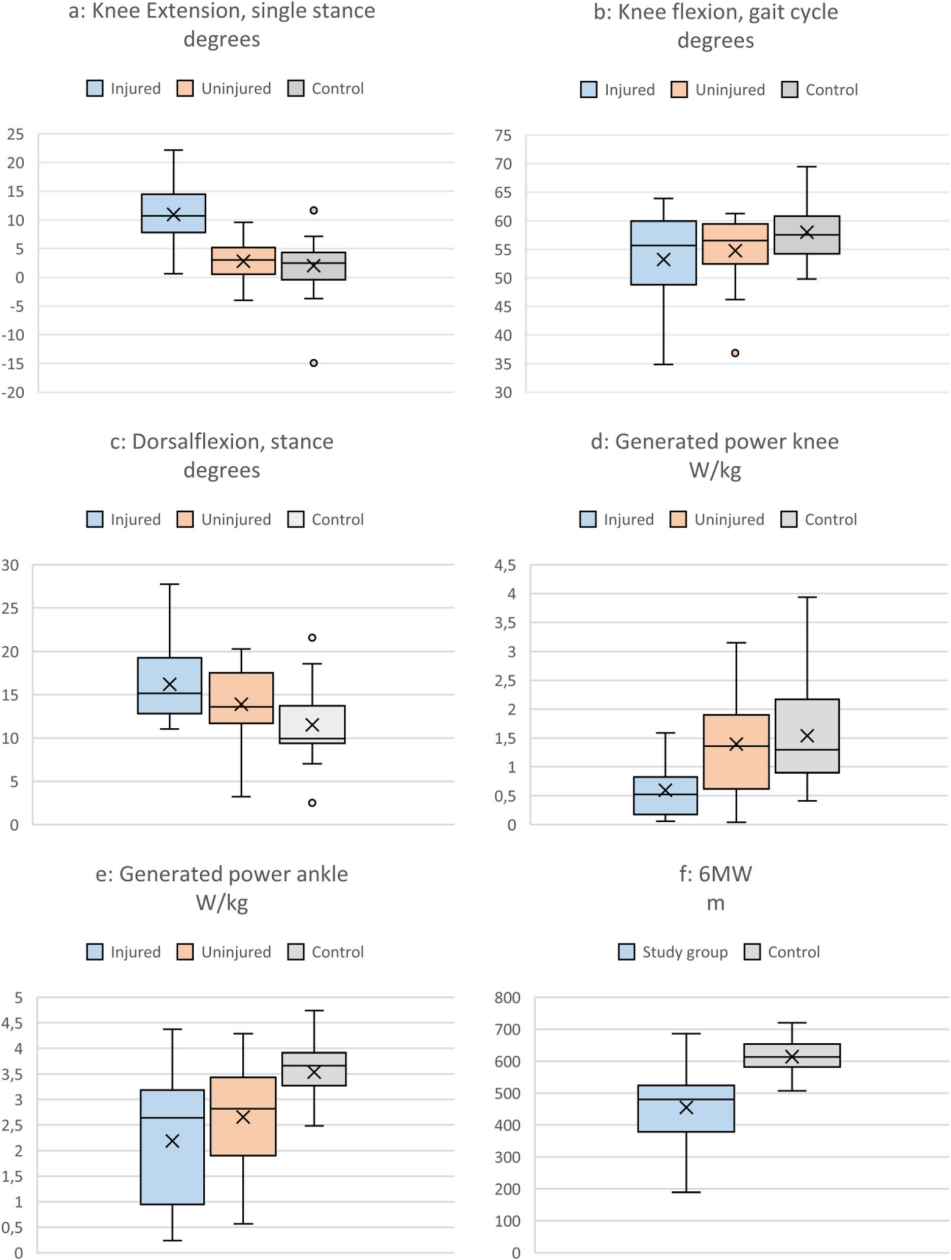
**a**-**f** Results presented in injured, uninjured and controls for variables **a:** Maximal knee extension during single stance; **b**: Maximal knee flexion during gait cycle; **c**: Maximal dorsiflexion during stance; **d**: Maximal generated power knee joint; **e**: Maximal generated power ankle joint; **f**: 6 MW: Numbers of meters on the 6 MW

Results from the instrumental gait analysis, ROM, pain estimation and 6 MW are presented in Table 3. Results from gait analysis and 6 MW are also presented in Fig. 3. In terms of ROM, 16 participants achieved knee flexion of more than 110°, while eight participants were unable to extend to 0°. No comparisons of ROM were made with a reference group. The smallest and largest changes in pain were 0 and 45 mm respectively before and after 6 MW. No comparisons of pain estimation were made with a reference group.

**Table 3:** Results from the gait analysis, passive range of motion, six minute walk-test and pain estimation

	**Injured side**		**Uninjured side**		**Reference group 1**		p**-value** **injured/uninjured**	*p***-value** **injured side/reference group 1**
**Gait analysis**								
	**Median**	**Range**	**Median**	**Range**	**Median**	**Range**		
Speed *m/s*	1.1	0.34–1.44	–	–	1.3	1.1–1.72	–	< 0.001*
Stance time *%*	61	59–69	64	61–78	61	58–63	< 0.001*	0.229
Step length *meters*	0.59	0.33–0.79	0.59	0.29–0.77	0.69	0.6–0.87	0.014*	0.001*
Knee flexion, (gait cycle) *degrees*	56	35–64	57	36–61	58	50–69	0.332	0.213
Knee extension, (single stance) *degrees*	- 11	(−22) – (−4)	- 3	(−9) - 6	- 2	(−15)-13	< 0.001*	< 0.001*
Ankle dorsiflexion, (stance) *degrees*	15	11–28	14	3–20	10	3–22	0.012*	0.002*
Max generating power knee (stance) *W/kg*	0.52	0.06–1.59	1.36	0.04–3.15	1.30	0.41–3.94	< 0.001*	< 0.001*
Max generating power ankle (stance) *W/kg*	2.63	0.24–4.38	2.82	0.56–4.29	3.66	2.48–4.74	0.023*	< 0.001*
**Passive range of motion**								
Knee flexion *degrees*	135	60–145	143	130–155	–	–	< 0.001*	–
Knee extension *degrees*	0	(−15) -10	0	0–10	–	–	0.005*	–
Ankle dorsiflexion *degrees*	17	5–30	20	10–30	–	–	0.006*	–
	**Study group**				**Reference group 2**			***p*-value** **injured side/reference group 2**
**Six-minute walk** *meters*	480	190–686	–	–	613	508–720	–	< 0.001*
**Pain** *VAS 0–100*	**Pre 6 MW**		**Post 6 MW**				**p-value** **pre/post**	
	2.5	0–65	15	0–85	–	–	0.001*	

*P*-value with one * means that the significant difference is less than *p* < 0.05.

## Discussion

The purpose of the study was to examine the walking ability of patients with tibial plateau fractures 3 months after the injury and to compare the injured side with the uninjured side, as well as with a healthy reference group. The hypothesis was that the walking pattern would be affected in patients after a TPF compared with a healthy population and that patients would have less joint mobility and more pain than an age- and gender-matched healthy population.

The most important findings in the present study were that the participants walked with an extension deficit and also had a detectable extension deficit in the knee joint during passive range of motion measurements.

The KOOS showed the largest differences in terms of the domains of symptoms, sport and quality of life compared with a healthy population. The domains of pain and ADL are of less value to the patients 3 months after the injury. Previous studies have shown that a TPF score of 68–83 points [15, 34] 1 year after injury and 60–88 points after 5 years [6, 9] indicates an ability to further improve over time. In a normal population aged 18–54 years, the score for pain was 97.2, symptoms 92.9–95.8, ADL 99–100, leisure/sport 88–95 and quality of life 88–94 [35].

The results from the ROM measurements are generally good. The average knee flexion was 135° after 3 months. Sixteen of the participants in the present study were able to flex their knee more than 110° and 18 were able to flex above 100°. The mean flexion on the injured side was still significantly less in comparison with the uninjured side. Jansen et al. and Elsoe et al. have reported approximately 125° knee flexion at 5 years after B and C fractures [6, 9]. This shows that participants in the present study already had a good ability to flex their knee at the early stage of rehabilitation. In the present study, eight participants were unable to extend their knees to 0°. Other studies have also presented similar residual extension deficits. According to Gaston, 62% of patients with B and C fractures had an extension deficit of less than 5° after 3 months, while, at 1 year, 21% still had an extension deficit greater than 5° [8].

The results of the instrumental gait analysis revealed differences in gait pattern such as shorter step length and shorter stance time on the injured side compared with the uninjured side. These results are in agreement with Warschawski et al., who also presented slower walking speed and shorter step length [14].

One of the most interesting findings was the difference between the instrumental gait analyses, which shows more extension deficit in the knee joint while walking compared with the extension deficit from passive range of motion. In the present study, we were, however, unable to ascertain why the participants had a greater extension deficit in the gait analysis than they had in ROM testing. One explanation could be that the pain increases during stance with an increased load and stops the participant from fully extending his/her leg. Moreover, reduced strength in the quadriceps muscle and a different pattern of movement could also affect the ability to extend the knee during stance. The extension deficit is probably one reason for the low generated power in the injured knee joint, only 37% compared with the contralateral, uninjured side. As mentioned in the method section, joint power depends on angular velocity and GRF. In a healthy person, there is a dynamic movement between flexion and extension during the stance phase [36]. People with an extension deficit in the knee joint have less movement between flexion and extension and there is for obvious reason lower angular velocity contributing to generated power. The study group also walked with a slower pace, which affects the angular velocity and by extension the generated power in comparison with the healthy reference group.

Because extension deficit is persistent in patients with TPF, it is still true that extension training is important to avoid the risk of future flexion contracture. It is also desirable to recover a normal walking pattern as quickly as possible, as walking with flexed knees loads the knee joints differently and affects the movement pattern in adjacent joints. Walking with a slightly flexed knee increases and alters the muscular activity around the joint [37] and may lead to an increased load on different parts of the joint surface, which might increase the risk of joint imbalance, as well as the risk of osteoarthritis in the long term.

The results from present study correspond well with the clinical experience of the patient group, with clinical signs of limping and compensation for loading on the injured leg. In the present study measurements of both the injured and uninjured sides were included in the analysis. It is important to keep in mind that the uninjured leg cannot be considered normal as it compensates for the injured leg. It is, however, still of interest to present differences between the injured and uninjured leg together with a healthy control group to avoid interference of a compensatory motion pattern in the individual’s non injured leg affecting the gait asymmetries. Therefore, a healthy control group was used for comparisons with the injured leg.

The results of the instrumental gait analysis revealed differences in terms of kinematics and kinetics. The differences seen in the 6 MW, where the participants were asked to walk as far as possible during 6 min, which meant that they probably slightly increased their walking speed, are even more pronounced. Several study participants exhibited a worse limp after a few minutes of walking, which was not seen during the instrumental gait analysis. When assessing pain with the VAS, it was shown that a walking distance of approximately 500 m can be provocative and cause pain in the knee.

There are a number of limitations to the present study. The results of the instrumental gait analysis may be affected by incorrect marker placement, soft-tissue artefacts and the small study group. Among most of the study participants, the walking patterns changed slightly during the instrumental gait analysis in the gait laboratory compared with the 6 MW. As mentioned above, several study participants increased their walking speed during the 6 MW and a clear limp appeared after a few minutes’ walk. It might had been clearer if there was just one reference group, but this was not applicable in the present study.

With a larger study population, it would be possible to divide the study population into different subgroups; for example, into surgically and non-surgically treated patients or subgroups depending on the fracture type according to AO/OTA. Clinically, it was thought that patients with type-C fractures would experience a more pronounced impact on walking function compared with patients with type-B fractures, but we are unable to confirm this after this study, as we have not compared subgroups. However, in the AO/OTA classification, the degree of dislocation does not influence the classification of the fracture type. A type-C fracture may have only a minor dislocation, which means that, even though the fracture is fully intra-articular, it might heal in a good position if not severely dislocated or if a good position was achieved during surgery. As seen in the result section of this study, there are some signs of heterogeneity in the study population. For example, there is a wide spread in the numbers of days between the injury and the time of gait analysis. The reason for this is the restrictions relating to weight-bearing the patients were given by the surgeon. This is usually based on the severity of the fracture. The group of tibial plateau fractures is a non-homogeneous group of fractures and it demands individual ordinations, especially for the patients with more severe fractures. In the study context, it is difficult if not impossible to collect a fully homogeneous study group, taking account of fractures of different types and the degree of dislocation, as well as the individual parameters in the in-depth study patient.

The study population in the present study was limited, but the result provides new knowledge not previously reported. It is of great interest to study the gait pattern using 3DGA in patients with different fracture types according to AO/OTA, as it may make it possible to crystallize a subgroup that one should be more vigilant with in the case of fracture treatment. It is probably of limited interest to assess all patients with 3DGA, but for some patients it provides a lot of detailed information not seen during an observational gait analysis. With a greater understanding on how joint angles and forces act around the knee joint, it is possible to deeply discuss a person’s problem after an injury or disease and use the information before continuing rehabilitation or make a more detailed decision before corrective surgery or other treatments. In the clinical setting, problems with altered range of motion, gait alterations and even limp are encountered in this group of patients. Only a limited number of studies have previously investigated physical function using the instrumental gait analysis of patients with TPF after surgical and non-surgical treatment [9, 14, 15]. By documenting the rehabilitation process from an early stage and following the patients over time, we might with greater certainty understand and focus on those patients who need increased rehabilitation efforts.

## Conclusion

Patients with tibial plateau fractures experience a considerable impact on their walking pattern 3 months after injury. These limitations are mainly related to the inability to extend the knee joint.

## Data Availability

The datasets used and/or analysed during the current study are available from the corresponding author in response to a reasonable request.

## Abbreviations

TPF: Tibial plateau fracture
SFR: Swedish Fracture Register
AO/OTA: Arbeitsgemeinschaft für Osteosynthesefragen/Orthopaedic Trauma Association
KOOS: Knee injury and Osteoarthritis Outcome Score (KOOS)
ROM: Passive range of motion
6 MW: Six-minute walking test
VAS: Visual Analogue Scale
